# Changes in left ventricular repolarization after short-term testosterone replacement therapy in hypogonadal males

**DOI:** 10.1007/s40618-019-01026-5

**Published:** 2019-03-05

**Authors:** G. Piccirillo, F. Moscucci, R. Pofi, G. D’Alessandro, M. Minnetti, A. M. Isidori, D. Francomano, A. Lenzi, P. E. Puddu, J. Alexandre, D. Magrì, A. Aversa

**Affiliations:** 1 Dipartimento di Scienze Cardiovascolari, Respiratorie, Nefrologiche, Anestesiologiche e Geriatriche, Policlinico Umberto I, “La Sapienza” University of Rome, Viale del Policlinico, 00185 Rome, Italy; 2 Department of Experimental Medicine, Sapienza University of Rome, Rome, Italy; 3 Division of Internal Medicine and Endocrinology, Madonna delle Grazie Hospital, Velletri, Rome, Italy; 4 EA 4650, Signalisation, électrophysiologie et imagerie des lésions d’ischémie reperfusion myocardique, Université de Caen, Normandie, France; 5 Department of Pharmacology, CHU Caen, Caen, France; 6 Dipartimento di Medicina Clinica e Molecolare, S. Andrea Hospital, “Sapienza” University of Rome, Rome, Italy; 7 Department of Experimental and Clinical Medicine, University of Catanzaro « Magna Grecia », Catanzaro, Italy

**Keywords:** Ventricular repolarization, QT, Sudden cardiac death, Hypogonadism, Testosterone, Androgen

## Abstract

**Background and aim:**

Evidences suggest that androgen deficiency is associated with sudden cardiac death (SCD). Our purpose was to analyse some electrocardiographic (ECG) markers of repolarization phase in hypogonadal patients either at baseline or after testosterone replacement therapy (TRT).

**Patients and Methods:**

Baseline and after 6 months of testosterone replacement therapy, 14 hypogonadal patients and 10 age-matched controls underwent a short-term ECG recordings at rest and immediately after a maximal exercise test. The following ECG parameters have been collected: QTe (the interval between the q wave the end of T wave), QTp (the interval between the q wave and the peak of T wave), and Te (the interval between the peak and the end of T wave).

**Results:**

At baseline, in the hypogonadal patients, corrected QTe and QTp values were longer at rest than in the controls at rest (*p* < 0.05), whereas, during the recovery phase, only the QTp remained significantly longer (*p* < 0.05). After TRT, hypogonadal patients showed an improvement only in Te (*p* < 0.05). Conversely, any difference between hypogonadal patients and control subjects was found with respect to the markers of temporal dispersion of repolarization phases, except for a worse QTp → Te coherence (*p* = 0.001) obtained during the recovery phase.

**Conclusions:**

In conclusion, at rest, hypogonadal patients suffer from a stable increase in the myocardial repolarization phase without an increase in its temporal dispersion and, hence, the SCD risk seems to be low.

## Introduction

Male hypogonadism is characterized by the presence of clinical symptoms of androgen deficiency (e.g. erectile dysfunction, delayed puberty, etc.) associated with low testosterone levels (< 12 nmol/L) [1]. This condition affects 6–12% of men aged between 40 and 69 years and it is strongly associated with cardiovascular disorders. Uncertain data are available about the effects of testosterone replacement therapy (TRT) on cardiovascular risk. The RHYME study clearly concludes that hypogonadal men receiving TRT did not show an increased cardiovascular risk [2]. Moreover, Corona et al. in a systematic review and misanalysis did not find a causal role between TRT and cardiovascular events [3]. Recently, it has been reported that low level of testosterone is associated with higher risk of sudden cardiac death (SCD) most likely due to a worsening in the myocardial repolarization phase [1, 4]. Supporting the hypothesis, gender- and hormone-related differences in myocardial repolarization phase length have been described and, hence, in the ECG-surface-derived QT interval. Indeed, in healthy condition, adult female subjects show a QT interval corrected for the heart rate significantly longer than male, this difference being absent before the puberty with a progressive QT interval shortening from 9 to 50 years old in male [5–10]. The abovementioned trend is thought to be related to progressive androgen level increase and, consistently, the opposite trend (i.e. QT interval increase) is detectable after the 60 years old [6–8]. Furthermore, the males after orchiectomy show QT interval longer than healthy age-matched male subjects and, even, the masculinized females have QT interval shorter than the normal ones. Moreover, the abuse of androgenic steroid in athletes is known to be related to the sudden cardiac death [11]. In this context, the analysis of temporal dispersion of myocardial repolarization might help in understanding some mechanisms underlying the impact of testosterone on arrhythmia propensity. Indeed, the myocardial repolarization phase, non-invasively studied on the surface electrocardiogram (ECG) by means of different QT segments measurement, short-term QT segment variability [12], QT/RR slope and QT-RR spectral coherence [12–17], yet imposed itself as a non-invasive marker of sudden cardiac death (SCD) risk in several cardiovascular and not cardiovascular conditions [12, 13, 18–21].

Therefore, the present experimental study sought to investigate non-invasively the myocardial repolarization phase and its temporal dispersion in a series of hypogonadal male patients either at baseline or after testosterone replacement therapy. All the ECG-derived parameters were studied both at rest and immediately after a maximal exercise test. Particularly, the evaluation of the ECG-derived parameters in the post-exercise phase aimed to study the repolarization in the absence of excessive muscle interferences, yet during intense autonomic nervous system imbalance [22–24] characterized by high vagal and sympathetic activity due to recovery from intense exercise.

## Methods

### Patients and protocol

To test the hypothesis of the testosterone influence on the left ventricular repolarization, we planned a single-center pilot prospective study. The diagnosis of hypogonadism was based on the presence of clinical symptoms related to this condition (e.g. reduced libido or erectile dysfunction) and on the results of standard hormonal exams (total testosterone < 12 nmol/L). After 6 months of enrollment time, we selected 14 subjects with hypogonadism candidates to testosterone replacement therapy (TRT) and 10 eugonadal age-matched controls. Particularly, nine patients had post-surgical hypogonadotropic hypogonadism (nine pituitary adenomas), one post-surgical hypogonadism (testicular cancer), two patients had idiopathic congenital hypogonadotropic hypogonadism and two had naïve Kallmann syndrome (hypogonadotropic hypogonadism and anosmia). Patients with hypogonadism and controls underwent a complete myocardial repolarization phase non-invasive study at baseline and after 6 months from the first administration of testosterone undecanoate injectable (1000 mg i.m injection repeated time 0 and after 6 weeks as indicated by product indication schedule). Blood samples were collected at baseline: the testosterone level was measured before the repolarization studies and at the end of study observation period, after 6 months of follow-up (total of three injections). All blood samples were collected by venipuncture in fasting patients; serum concentrations of testosterone were measured by chemiluminescence.

The clinical assessment included physical examination, echocardiogram, 5 min of single-lead (D II) ECG recording at rest in supine position and 10 min of single-lead ECG recordings during the post-exercise recovery phase in sitting position on the bike. All subjects underwent Bruce protocol stress testing; patients with typical angina were excluded from the study as well as those with a ECG responses characterized by 1 mm or more horizontal or downsloping ST segment depression, measured at 80 ms sec after the J point. Tests were considered valid only if the subject reached at least 85% of the maximal age-corrected heart rate. All ECG registrations were collected baseline, and after 6 months from commencement of TRT.

### Data processing

We used a custom-designed card (National Instruments USB-6008; National Instruments, Austin, TX, USA) to acquire and digitalize the ECG signals; the sampling frequency was 500 Hz. Points used for the ECG segment analysis were detected automatically by a classic adaptive derivative/threshold algorithm. We designed and produced a software for data acquisition, storage, and analysis with the LabView program (National Instruments). After a linear interpolation, an expert cardiologist (GP) checked the different points and, when needed, manually corrected the mistakes with an interactive software [15, 16, 22, 25–27]. All ECG data were analyzed in a single-blind fashion.

**Fig. 1:**
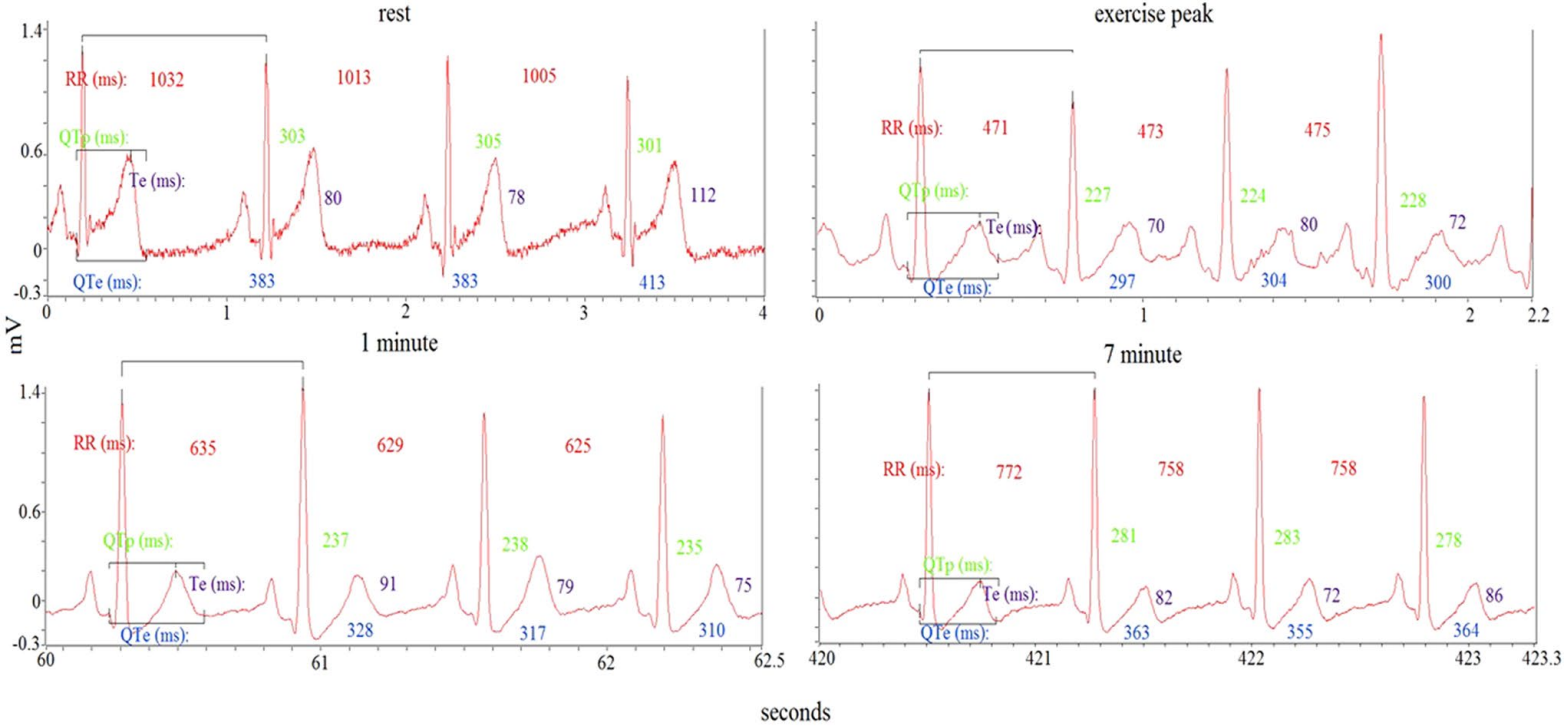
Representative example of RR, QTe, QTp, and Te interval measurements from a single-lead ECG at rest, during the peak, the first and the 10th min of exercise recovery

**Fig. 2:**
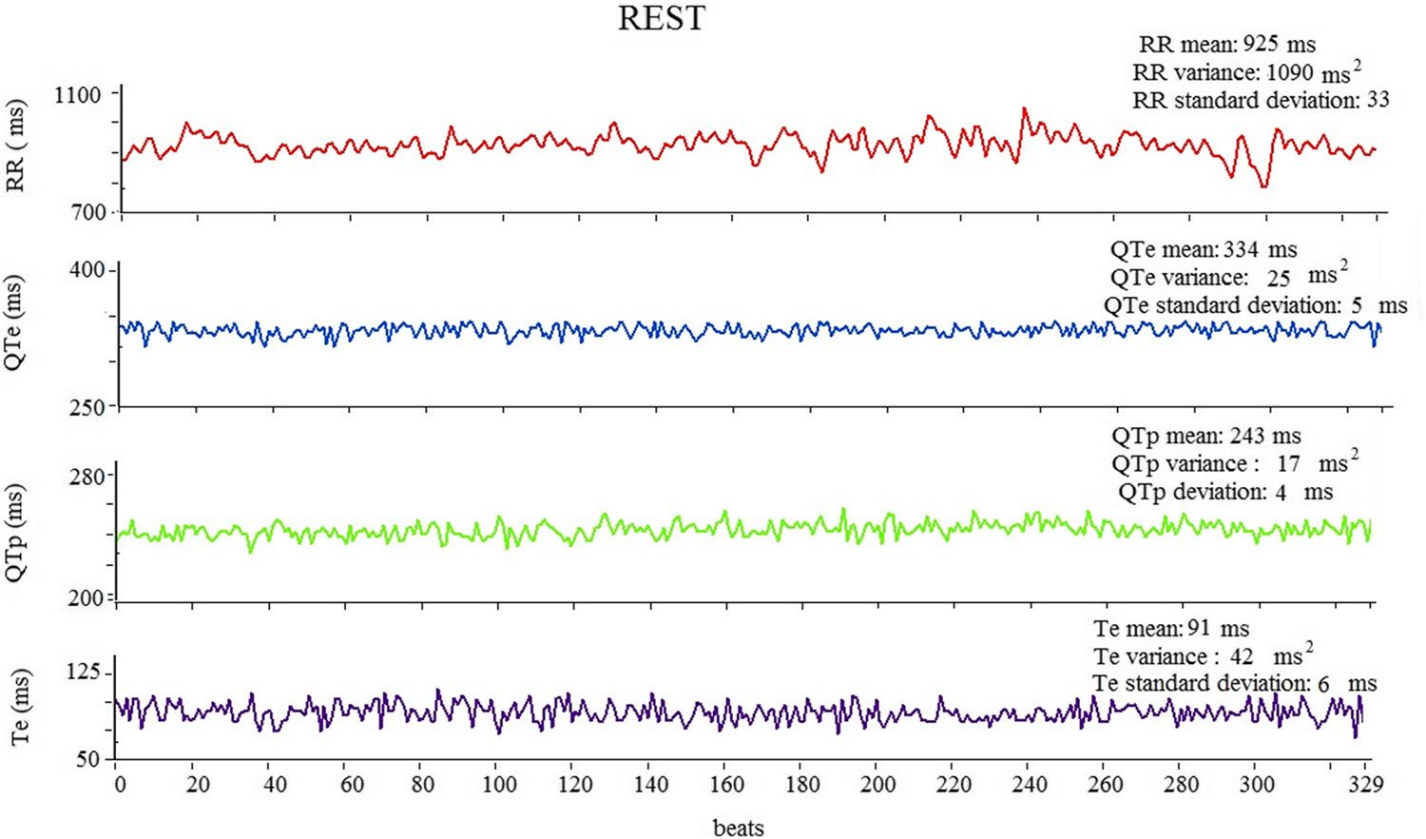
Representative example of a 5-min ECG recording and derived variables on RR, QTe, QTp, and Te intervals at rest

**Fig. 3:**
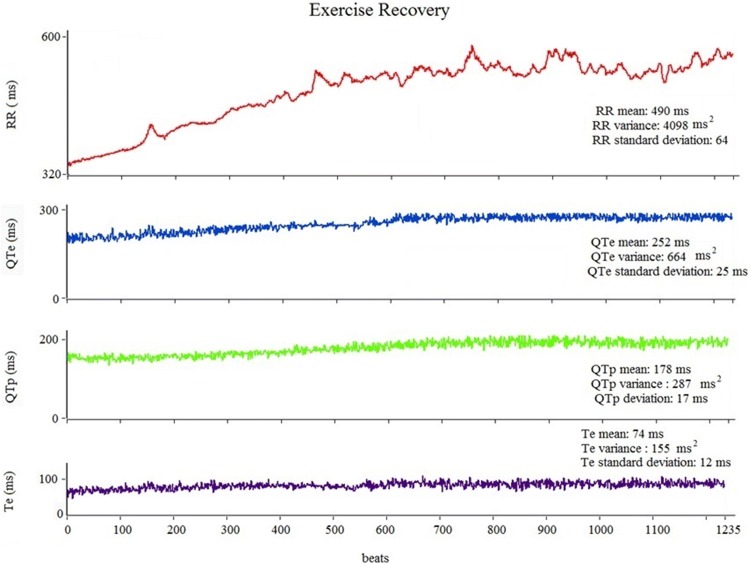
Representative example of a 10-min ECG recording and derived variables on RR, QTe, QTp, and Te intervals during recovery exercise

Beat-to-beat ECG intervals obtained at rest and during exercise recovery were: RR, QTe (the interval between the q wave the end of T wave), QTp (the interval between the q wave and the peak of T wave), and Te (the interval between the peak and the end of T wave) [15, 16, 22, 25–28] (Fig. 1). We, therefore, calculated mean and variance values for each of these intervals and then we used the original formula proposed by Berger et al. [29] to calculate three different QT variability indexes [15, 16, 22, 25–27] (Figs. 2, 3):

$$ \begin{aligned} {\text{QT}}_{\text{e}} {\text{VI}} & = { \log }_{ 10} \left\{ {\left[ {\left[ {{\text{QT}}_{\text{e}} {\text{variance}}} \right]/} \right[{\text{QT}}_{\text{e}} {\text{mean}}\left] {^{ 2} } \right]/\left[ {\left[ {\text{RRvariance}} \right]/} \right[{\text{RRmean}}]^{ 2} ]} \right\} \\ {\text{QT}}_{\text{p}} {\text{VI}} & = { \log }_{ 10} \left\{ {\left[ {\left[ {{\text{QT}}_{\text{p}} {\text{variance}}} \right]/} \right[{\text{QT}}_{\text{p}} {\text{mean}}\left] {^{ 2} } \right]/\left[ {\left[ {\text{RRvariance}} \right]/} \right[{\text{RRmean}}]^{ 2} ]} \right\} \\ {\text{T}}_{\text{e}} {\text{VI}} & = { \log }_{ 10} \left\{ {\left[ {\left[ {{\text{T}}_{\text{e}} {\text{variance}}} \right]/} \right[{\text{T}}_{\text{e}} {\text{mean}}\left] {^{ 2} } \right]/\left[ {\left[ {\text{RRvariance}} \right]/} \right[{\text{RRmean}}]^{ 2} ]} \right\}. \\ \end{aligned} $$

**Fig. 4:**
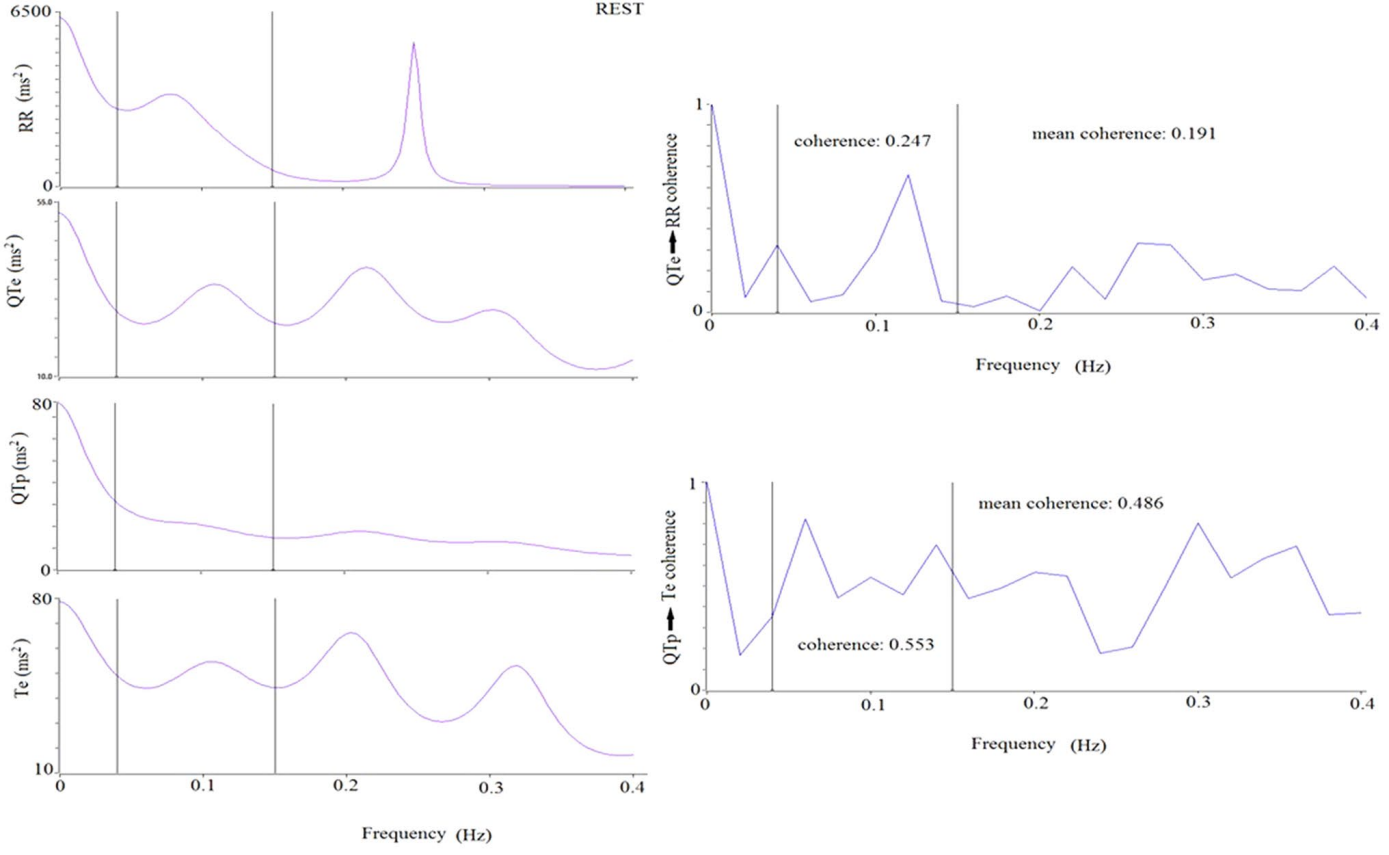
Representative example of a 5-min ECG recording power spectral analysis (left panels) and related coherence (right panels) at rest

The same ECG intervals were also used for power spectral (autoregressive algorithm) and cross-spectral analysis (Fig. 4). Cross-spectral analysis indicated the influences on the different oscillations (coherence function) between RR, QTe, QTp and Te [12–16, 22, 25–27] (Fig. 4). Coherence expresses an index (from 0 to 1) of a linear association between the two signals [12–16, 22, 25–27] (Fig. 4).

**Fig. 5:**
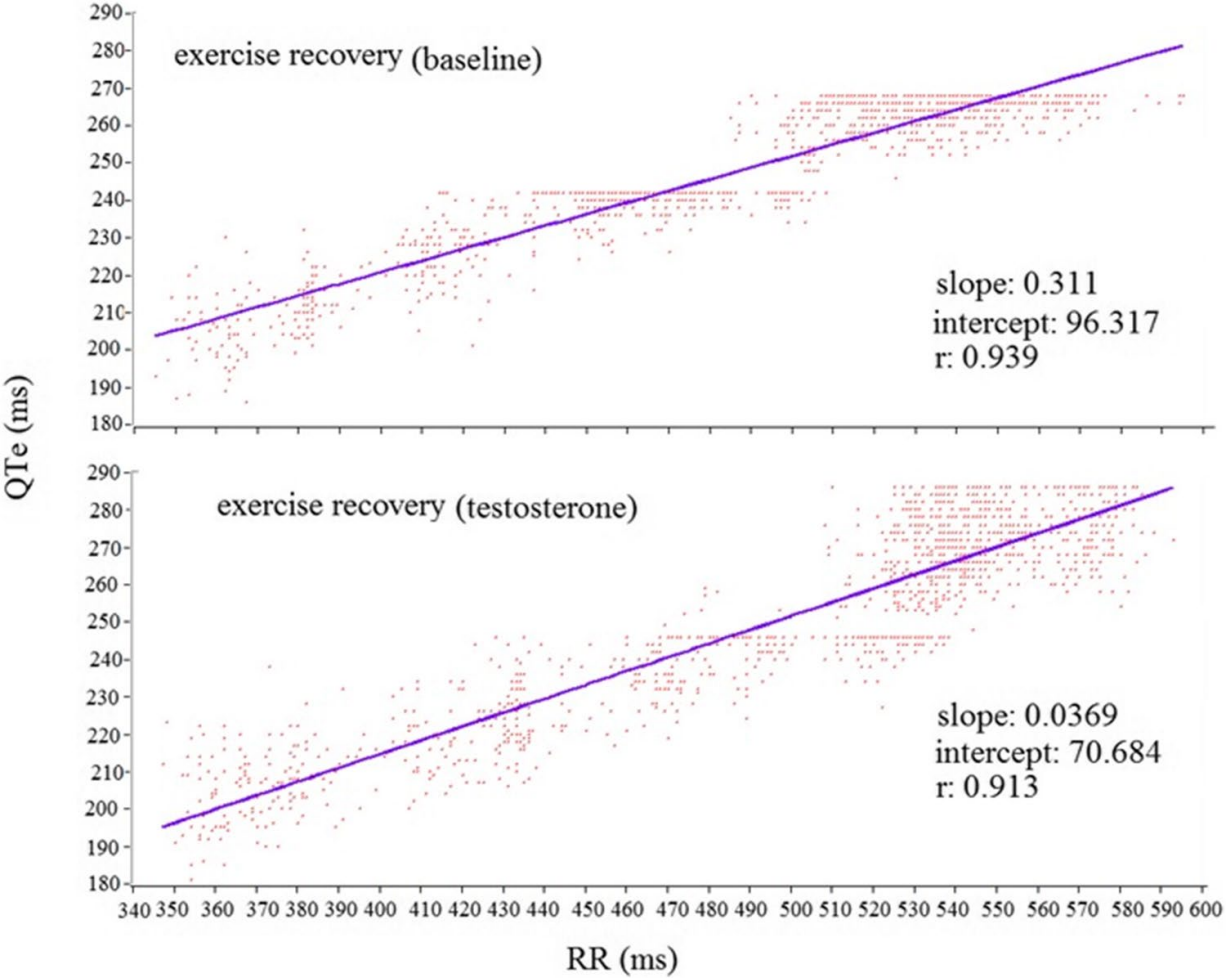
Representative example of QTe-RR slope during exercise recovery in baseline and after replacement therapy

Linear regression was used to calculate QTe-RR, QTp-RR and Te-RR slopes (Fig. 5) [14, 30–33]. This analysis was conducted only during exercise recovery because in rest the number of QT was not sufficient for significant linear regression [30].

From the ECG segments, the QTe, QTp, and Te intervals were corrected by the Bazett (QTe/RR^0.5^; QTp/RR^0.5^; Te/RR^0.5^), Fridericia (QTe/RR^0.33^; QTp/RR^0.33^; Te/RR^0.33^), Lilly (QTe/RR^0.4^; QTp/RR^0.4^; Te/RR^0.4^), and Framingham (QTe + [0.154 × {1000 − RR}]; QTp + [0.154 × {1000 − RR}]; Te + [0.154 × {1000 − RR}]) [15, 33] formulas. We calculated the repolarization corrected variables on the ECG overall length recordings at rest and during the recovery phase both at baseline and after testosterone replacement therapy. Moreover, we calculated manually the instantaneous corrected repolarization variables on three consecutive RR and on the following QRS-T (QTe, QTp and Te) intervals during the first minute at rest, at the exercise peak and, also, at the 1st, 3rd, 5th, 7th, 9th minutes during exercise recovery [24] with tangential method and using the ECG II lead. In particular, we measured the QTe interval as the time between QRS onset and the point at which the isoelectric line intersected a tangential line drawn at the maximal downslope of the positive *T* wave; instead the QTp was obtained measuring the interval from *q* and peak of *T* wave; finally Te interval was the difference between QTe and QTp. On the contrary, the QTe, QTp and Te data, obtained on baseline and after exercise recovery, were collected during the two whole recordings with the previous described and cited software. Finally, due the change of position (during supine or cycle) of patients could affect the amplitude and consequently the end of *T* wave, we checked possible variation of voltage of *T* wave [12].

### Statistical analysis

We reported data as mean ± SD or as interquartile range, respectively, for normal and skewed distribution data. We used Student’s *t* test to compare data for the normally distributed variables; on the contrary, we used Mann–Whitney to compare non-normally distributed variables (as evaluated by Kolgomorov–Smirnov test). We used the paired *t* test, for the normally distributed variables, and Wilcoxon test, for non-normally distributed variables, to compare data during baseline and replacement therapy. We considered statistically significant *p* values < 0.05. For statistical analysis, we used SPSS-PC + [SPSS-PC + Inc, Chicago, Illinois].

## Results

During TRT one patient interrupted the therapy for dysuria.

At baseline, general characteristics and echocardiographic data were similar between hypogonadal patients and normal subjects (Table 1). Furthermore, no difference was found regarding exercise data or voltage of *T* wave (Table 1). Naturally, testosterone levels were significant lower in the hypogonadal patients. Significant changes in PSA, hematocrit and waist circumference were reported in patients after 6 months testosterone undecanoate treatment (*p* < 0.05, data not shown) according to our previously published data obtained in hypogonadal men [34].

**Table 1:** General characteristic of the two study groups

Variables	Hypogonadal subjects *N* = 14	Control subjects *N* = 10	*P* values *t* test
Age (years)	54 ± 16	48 ± 13	Ns
BMI (kg/m^2)^	28 ± 3.5	27 ± 3.9	Ns
Waist circ. (cm)	98 ± 1.3	97 ± 1.7	Ns
LVEF (%)	61 ± 6	62 ± 5	Ns
LVMI (g/m^2^)	97 ± 11	89 ± 12	Ns
HR peak (b/m)	122 ± 34	130 ± 28	Ns
SBP peak (mm Hg)	173 ± 17	167 ± 21	Ns
Peak workload (W)	121 ± 34	130 ± 28	Ns
Exercise duration (min)	15 ± 4	16 ± 4	Ns
Rate pressure product	24,910 ± 5248	26,373 ± 5255	Ns
Testosterone (nmol/L)	3.97 ± 3.26	17.73 ± 3.89	< 0.001
PSA (ng/dL)	0.91 ± 0.26	2.73 ± 0.79	< 0.001
Hematocrit	42.7 ± 0.3	43.9 ± 0.9	Ns

*BMI* body mass index, *LVEF* left ventricular ejection fraction, *LVMI* left ventricular mass index, *HR* hear ratio, *SBP* systolic blood pressure

**Fig. 6:**
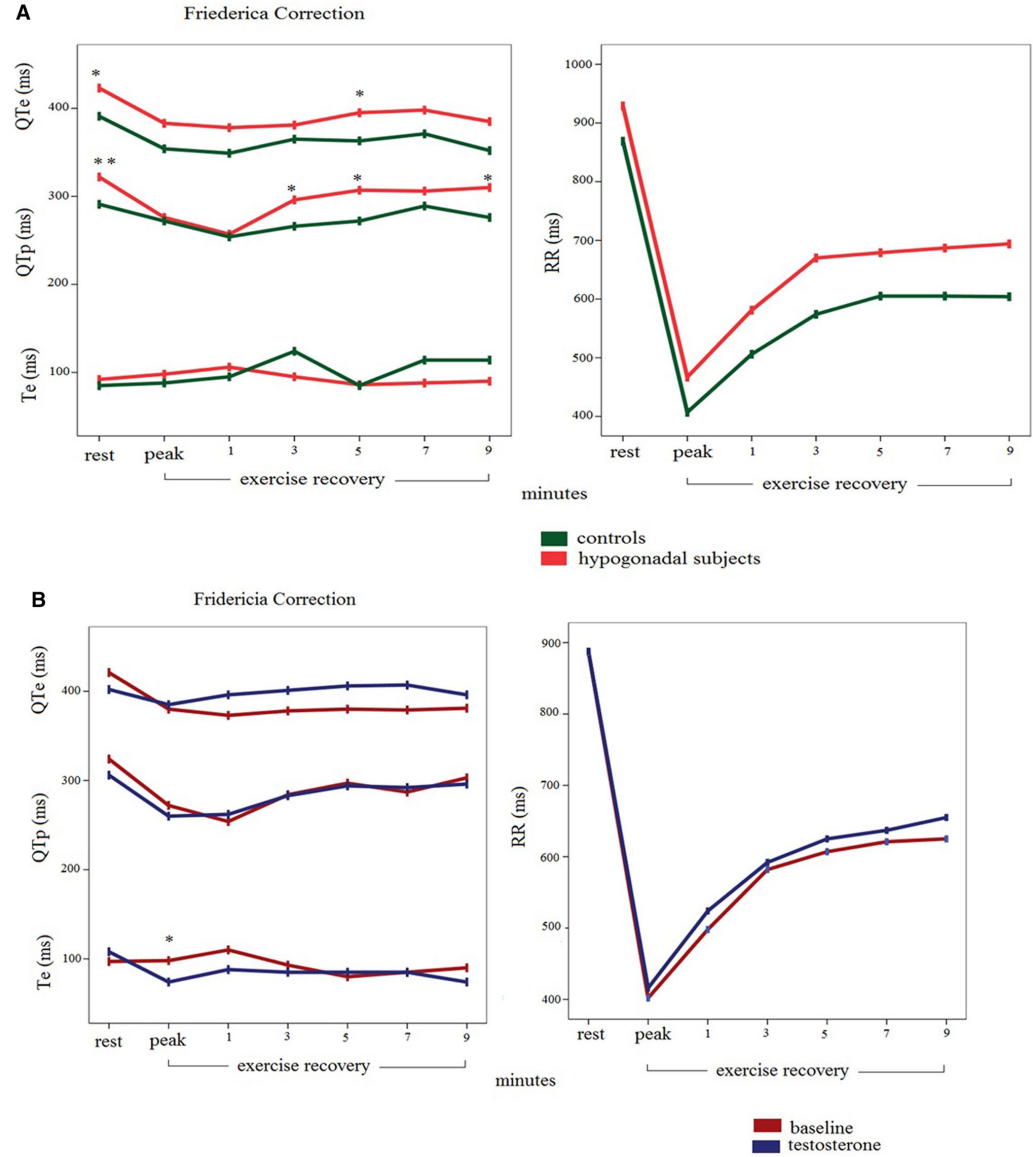
**a** QTe, QTp, and Te with Fridericia correction and RR intervals at rest, during the peak, the first and the 10th minute of exercise recovery in hypogonadal (red) and control subjects (green). *p < 0.05 or **p < 0.001: hypogonadal patients vs controls.** b** QTe, QTp, and Te with Fridericia correction and RR intervals at rest, during the peak, the first and the 10th minute of exercise recovery in hypogonadal (baseline) and during replacement therapy (blue) in hypogonadal subjects. *p < 0.05 baseline vs control. These data are detected manually with tangential method

QTe and QTp were significantly longer in hypogonadal patients at rest (Table 2). QTe, but not QTe_Bazett_ (for an overestimation of Bazett’s formula during exercise), showed similar behavior during the recovery phase exercise recovery (Table 2). As regards, the instantaneous repolarization variables, the corrected and raw QTe measurements were significantly longer in hypogonadal patients only at rest at the 5th minute of the recovery phase (Table 3, Fig. 6a). Instead, QTp was often significantly longer, regardless the heart rate. In fact at rest, during the 3rd, 5th and 9th minutes QTp were longer in the hypogonadal patients (Table 3, Fig. 6a) than controls, but this behavior was not reported at the exercise peak and during the 1st minute when the heart rate was the highest.

**Table 2:** QTe, QTp and Te data at baseline and after exercise

Variables	Hypogonadal subjects *N* = 14	Control subjects *N* = 10	*P* values
Rest			
HR (beats/min)	67 ± 11	72 ± 15	Ns
QTe (ms)	410 ± 32	371 ± 27	0.004
QTe_Bazett_ (ms)	430 ± 33	402 ± 25	0.032
QTe_Fridericia_ (ms)	421 ± 27	391 ± 15	0.002
QTe_Lilly_ (ms)	426 ± 29	395 ± 18	0.007
QTe_Framingham_ (ms)	422 ± 28	391 ± 17	0.005
QTp (ms)	314 ± 23	276 ± 23	0.001
QTp_Bazett_ (ms)	323 ± 20	299 ± 16	0.003
QTp_Fridericia_ (ms)	322 ± 20	291 ± 13	0.000
QTp_Lilly_ (ms)	325 ± 21	294 ± 12	0.000
QTp_Framingham_ (ms)	325 ± 26	296 ± 16	0.005
Te (ms)	97 ± 14	94 ± 11	Ns
Te_Bazett_ (ms)	102 ± 14	103 ± 15	Ns
Te_Fridericia_ (ms)	99 ± 13	100 ± 13	Ns
Te_Lilly_ (ms)	101 ± 14	101 ± 13	Ns
Te_Framingham_ (ms)	108 ± 28	115 ± 30	Ns
10 min exercise recovery			
HR (beats/min)	96 ± 19	109 ± 20	Ns
QTe (ms)s	306 ± 36	273 ± 27	0.022
QTe_Bazett_ (ms)	382 ± 21	366 ± 25	Ns
QTe_Fridericia_ (ms)	354 ± 23	332 ± 22	0.023
QTe_Lilly_ (ms)	365 ± 21	344 ± 23	0.035
QTe_Framingham_ (ms)	360 ± 21	340 ± 19	0.026
QTp (ms)	227 ± 30	204 ± 24	Ns
QTp_Bazett_ (ms)	283 ± 19	273 ± 23	Ns
QTp_Fridericia_ (ms)	263 ± 21	248 ± 22	Ns
QTp_Lilly_ (ms)	271 ± 20	258 ± 22	Ns
QTp_Framingham_ (ms)	281 ± 17	271 ± 18	Ns
Te (ms)	79 ± 10	70 ± 12	Ns
Te_Bazett_ (ms)	99 ± 11	94 ± 17	Ns
Te_Fridericia_ (ms)	92 ± 10	85 ± 15	Ns
Te_Lilly_ (ms)	94 ± 10	87 ± 16	Ns
Te_Framingham_ (ms)	132 ± 18	137 ± 19	Ns

These data are detected automatically with a custom software

*HR* heart ratio, *QTe* QT end, *QTp* QT peak, *Te* T end

**Table 3:** QTe, QTp and Te data at rest, at exercise peak and during 1, 3, 5, 7 and 9 min of recovery

	Rest	Exercise peak	1 min	3 min	5 min	7 min	9 min
Hypogonadal subjects							
RR (ms)	929 ± 204	467 ± 83	581 ± 120	670 ± 176	679 ± 128	687 ± 151	694 ± 44
QTe (ms)	401 ± 33*	297 ± 50	315 ± 41*	332 ± 47	346 ± 38*	351 ± 52*	339 ± 42*
QTe_Bazett_ (ms)	430 ± 33*	436 ± 57	416 ± 47	410 ± 44	422 ± 29*	425 ± 44	411 ± 41
QTe_Fridericia_ (ms)	423 ± 27*	383 ± 52	378 ± 41	381 ± 40	395 ± 29*	398 ± 44	385 ± 37
QTe_Lilly_ (ms)	426 ± 29*	404 ± 54	393 ± 42	392 ± 41	405 ± 28*	409 ± 44	395 ± 38
QTe_Framingham_ (ms)	422 ± 28*	379 ± 44	379 ± 32*	383 ± 37	396 ± 26*	399 ± 41	387 ± 33
Control subjects							
RR (ms)	869 ± 203	407 ± 50	506 ± 100	574 ± 110	605 ± 126	604 ± 106	603 ± 158
QTe (ms)	371 ± 27*	263 ± 47	278 ± 30*	303 ± 30	306 ± 28*	313 ± 23*	297 ± 53*
QTe_Bazett_ (ms)	402 ± 25*	411 ± 62	392 ± 28	402 ± 23	396 ± 16*	404 ± 17	384 ± 39
QTe_Fridericia_ (ms)	391 ± 15*	354 ± 56	349 ± 26	365 ± 21	363 ± 15*	371 ± 14	352 ± 43
QTe_Lilly_ (ms)	395 ± 18*	376 ± 58	366 ± 26	379 ± 21	376 ± 14*	384 ± 14	364 ± 42
QTe_Framingham_ (ms)	391 ± 17*	354 ± 43	354 ± 20*	368 ± 18	367 ± 13*	374 ± 12	358 ± 34
Hypogonadal subjects							
QTp (ms)	314 ± 23*	214 ± 23	214 ± 23	258 ± 34*	269 ± 33*	270 ± 45	273 ± 27*
QTp_Bazett_ (ms)	323 ± 20*	314 ± 16	283 ± 17	318 ± 22*	328 ± 28*	326 ± 34	331 ± 24*
QTp_Fridericia_ (ms)	322 ± 20**	276 ± 17	257 ± 16	296 ± 23*	307 ± 27*	306 ± 36	310 ± 20*
QTp_Lilly_ (ms)	325 ± 21**	291 ± 16	257 ± 16	304 ± 22*	315 ± 27*	314 ± 35	319 ± 21*
QTp_Framingham_ (ms)	325 ± 26*	296 ± 13	278 ± 13	309 ± 20*	744 ± 105	318 ± 30	321 ± 19*
Control subjects							
QTp (ms)	276 ± 23*	202 ± 32	202 ± 33	220 ± 25*	230 ± 30*	243 ± 25	233 ± 41*
QTp_Bazett_ (ms)	299 ± 16*	316 ± 41	285 ± 39	292 ± 22*	297 ± 30*	315 ± 29	301 ± 27*
QTp_Fridericia_ (ms)	291 ± 13*	272 ± 37	254 ± 35	266 ± 20*	272 ± 27*	289 ± 25	276 ± 32*
QTp_Lilly_ (ms)	294 ± 12*	289 ± 39	266 ± 37	276 ± 21*	282 ± 28*	299 ± 26	286 ± 30*
QTp_Framingham_ (ms)	296 ± 16**	293 ± 28	278 ± 27	286 ± 16*	279 ± 27*	304 ± 20	294 ± 22*
Hypogonadal subjects							
Te (ms)	79 ± 10	76 ± 17	88 ± 27	83 ± 10	75 ± 15	78 ± 10	78 ± 20
Te_Bazett_ (ms)	99 ± 11	112 ± 25	117 ± 42	103 ± 12	92 ± 17	94 ± 11	96 ± 28
Te_Fridericia_ (ms)	92 ± 10	98 ± 21	106 ± 35	95 ± 10	86 ± 16	88 ± 10	90 ± 25
Te_Lilly_ (ms)	94 ± 10	103 ± 23	110 ± 38	98 ± 11	88 ± 16	90 ± 10	92 ± 27
Te_Framingham_ (ms)	132 ± 18	158 ± 18	152 ± 32	133 ± 24	125 ± 19	126 ± 22	126 ± 33
Control subjects							
Te (ms)	70 ± 12	65 ± 16	76 ± 14	102 ± 65	96 ± 71	96 ± 73	96 ± 76
Te_Bazett_ (ms)	94 ± 17	102 ± 22	107 ± 18	137 ± 93	126 ± 98	124 ± 98	124 ± 100
Te_Fridericia_ (ms)	85 ± 15	88 ± 20	95 ± 16	124 ± 82	85 ± 16	114 ± 88	114 ± 91
Te_Lilly_ (ms)	87 ± 16	93 ± 21	100 ± 17	129 ± 86	119 ± 92	119 ± 92	118 ± 95
Te_Framingham_ (ms)	137 ± 19	157 ± 13	152 ± 18	167 ± 69	157 ± 75	157 ± 75	157 ± 79

These data are detected manually with tangential method. *p < 0.05 or **p < 0.001: hypogonadal patients vs controls

*RR* RR interval, *QTe* QT end, *QTp* QT peak, *Te* T end

**Fig. 7:**
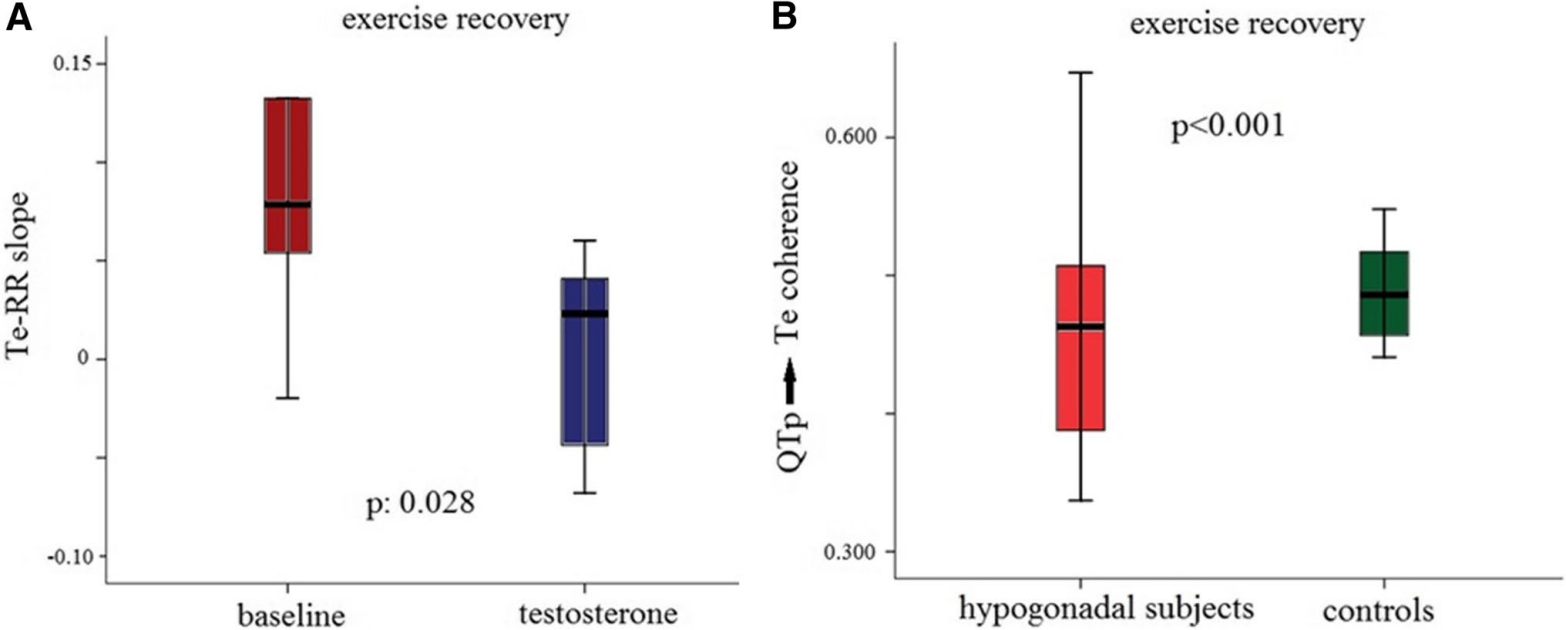
**a** QTp → Te during exercise recovery in hypogonadal and control subjects. In the box plots, the central line represents the median distribution. Each box spans from 25th to 75th percentile points, and error bars extend from 10th to 90th percentile points. **b** Te-RR slope during exercise recovery at baseline and replacement therapy in hypogonadal subjects. In the box plots, the central line represents the median distribution. Each box spans from 25th to 75th percentile points, and error bars extend from 10th to 90th percentile points. These data are detected automatically with a custom software

No significant differences were found with respect to the myocardial repolarization dispersion variables (Table 4), except for the QTp → Te coherence during the recovery phase (Table 4, Fig. 7a), where this parameter was lower in the hypogonadal subjects (*p* < 0.001).

**Table 4:** QTe, QTp and Te Variabilities and Coherence Data

Variables	Hypogonadal subjects *N* = 14	Control subjects *N* = 10	*P* values
Rest			
QTe mean (ms)	371 ± 36	336 ± 22	0.016
QTe variance (ms)^2^	47 [29]	38 [30]	Ns
QTe standard deviation	7 ± 1	6 ± 1	Ns
RR (ms)	924 ± 201	854 ± 187	Ns
RR variance (ms^2^)	766 [1975]	915 [1280]	Ns
RR standard deviation	37 ± 22	30 ± 11	Ns
QTeVI	− 0.57 [0.75]	− 0.72 [0.63]	Ns
RR → QTe, coherence	0.199 ± 0.029	0.214 ± 0.030	Ns
QTp mean (ms)	274 ± 27	242 ± 20	0.04
QTp variance (ms^2^)	30 [25]	21 [15]	Ns
QTp standard deviation (ms^2^)	5 ± 1	5 ± 1	Ns
QTpVI	− 1.20 [1.03]	− 1.33 [0.54]	Ns
RR → QTp, coherence	0.223 ± 0.028	0.209 ± 0.026	Ns
Te (ms)	97 ± 14	95 ± 11	Ns
Te variance (ms)	71 [52]	59 [42]	Ns
Te standard deviation	8 ± 2	7 ± 2	Ns
TeVI	− 0.004 [0.86]	− 0.186 [0.60]	Ns
RR → Te, coherence	0.212 ± 0.036	0.195 ± 0.025	Ns
QTp → Te, coherence	0.479 ± 0.049	0.462 ± 0.095	Ns
10 min exercise recovery			
QTe mean (ms)	306 ± 36	273 ± 27	0.022
QTe variance (ms^2^)	337 [571]	252 [261]	Ns
QTe standard deviation	25 ± 18	19 ± 6	Ns
RR (ms)	651 ± 141	565 ± 11	Ns
RR variance (ms^2^)	2456 [3000]	2716 [3875]	Ns
RR standard deviation	62 ± 39	57 ± 25	Ns
QTeVI	− 0.23 [0.57]	− 0.51 [0.57]	Ns
RR → QTe, coherence	0.226 ± 0.054	0.246 ± 0.060	Ns
QTe-RR slope,	0.27 [0.14]	0.25 [0.21]	Ns
QTp mean (ms)	227 ± 30	204 ± 24	Ns
QTp variance (ms^2^)	329 [380]	263 [817]	Ns
QTp standard deviation (ms^2^)	19 ± 6	16 ± 6	Ns
QTpVI	− 0.07 [0.41]	− 0.27 [0.73]	Ns
QTp-RR slope	0.29 [0.13]	0.21 [0.32]	Ns
RR → QTp, coherence	0.225 ± 0.042	0.226 ± 0.049	Ns
Te (ms)	79 ± 10	70 ± 12	Ns
Te variance (ms)	113 [82]	89 [82]	Ns
Te standard deviation	12 ± 5	10 ± 3	Ns
TeVI	0.50 [0.82]	0.06 [0.88]	Ns
RR → Te, coherence	0.208 ± 40	0.217 ± 0.038	Ns
QTp → Te, coherence	0.442 ± 0.083	0.569 ± 0.070	0.001
Te-RR slope,	0.041 [0.11]	0.036 [0.07]	Ns

These data are detected automatically with a custom software. Values are expressed as mean ± SD or median [interquartile range 75th percentile–25th percentile]

*QTe* QT end, *QTp* QT peak, *Te* T end

After the TRT, the serum testosterone level was significant higher than baseline (from 3.86 ± 4.10 to 13.12 nmol/L, *p* < 0.001). Other general data (BMI, LVEF, LVMI, heart rate, blood pressure) at rest and during exercise (heart rate peak, systolic blood pressure peak, peak workload, exercise duration and rate pressure product) did not change. As far as this period, at rest and during the recovery phase most of the repolarization data were not significantly different. Conversely, non-corrected QTe (*p* < 0.05) and QTp (*p* < 0.05) at rest, Te with all corrections (*p* < 0.05) at the heart rate peak (Fig. 6b; Table 5) and Te-RR slope (*p* < 0.05) during recovery (Fig. 7b; Table 6) were significantly reduced with respect the baseline. We have found no difference between control group and hypogonadal subjects during TRT in ECG data. Finally, for a better understanding of the reported data, the instantaneous QRS-T data showed in the Tables 3 and 5 and Fig. 6 are obtained manually; on the contrary, data reported in the Tables 2, 4 and 6 and Fig. 7 are obtained automatically with the previous cited custom software.

**Table 5:** QTe, QTp and Te Data at rest, at exercise peak and during 1,3,5,7 and 9 min of recovery after testosterone therapy

	Rest	Exercise peak	1 min	3 min	5 min	7 min	9 min
Baseline							
RR (ms)	887 ± 2	402 ± 57	498 ± 97	582 ± 168	607 ± 115	621 ± 142	625 ± 160
QTe (ms)	402 ± 36*	280 ± 27	294 ± 39	313 ± 27	321 ± 23	322 ± 26	324 ± 34
QTe_Bazett_ (ms)	431 ± 40	444 ± 40	421 ± 62	416 ± 28	414 ± 29	411 ± 25	414 ± 43
QTe_Fridericia_ (ms)	421 ± 34	380 ± 33	373 ± 51	378 ± 20	380 ± 23	379 ± 20	381 ± 36
QTe_Lilly_ (ms)	425 ± 36	405 ± 36	392 ± 55	393 ± 22	393 ± 25	391 ± 21	394 ± 38
QTe_Framingham_ (ms)	419 ± 33	372 ± 25	372 ± 38	378 ± 17	381 ± 18	380 ± 17	381 ± 30
Testosterone							
RR (ms)	888 ± 97	416 ± 54	524 ± 86	592 ± 78	625 ± 89	637 ± 87	655 ± 90
QTe (ms)	378 ± 29*	248 ± 28	285 ± 18	308 ± 21	320 ± 21	323 ± 21	320 ± 20
QTe_Bazett_ (ms)	402 ± 29	405 ± 17	396 ± 17	401 ± 15	406 ± 25	407 ± 27	396 ± 20
QTe_Fridericia_ (ms)	394 ± 27	332 ± 31	355 ± 12	367 ± 15	375 ± 21	377 ± 21	369 ± 17
QTe_Lilly_ (ms)	397 ± 27	352 ± 32	371 ± 13	380 ± 14	387 ± 22	389 ± 23	379 ± 17
QTe_Framingham_ (ms)	395 ± 26	332 ± 31	320 ± 13	371 ± 13	378 ± 18	379 ± 18	373 ± 15
Baseline							
QTp (ms)	309 ± 26**	201 ± 18	201 ± 18	236 ± 25	251 ± 25	245 ± 35	258 ± 24
QTp_Bazett_ (ms)	332 ± 31	317 ± 14	286 ± 13	312 ± 20	324 ± 31	311 ± 28	329 ± 27
QTp_Fridericia_ (ms)	324 ± 25	272 ± 14	254 ± 12	284 ± 17	297 ± 26	287 ± 29	303 ± 22
QTp_Lilly_ (ms)	327 ± 27	290 ± 14	266 ± 11	295 ± 17	307 ± 28	301 ± 24	313 ± 23
QTp_Framingham_ (ms)	327 ± 30	293 ± 11	278 ± 8	300 ± 15	311 ± 21	303 ± 22	316 ± 20
Testosterone							
QTp (ms)	194 ± 22**	194 ± 22	211 ± 20	237 ± 16	251 ± 17	251 ± 26	257 ± 17
QTp_Bazett_ (ms)	312 ± 20	302 ± 26	293 ± 26	310 ± 20	319 ± 25	316 ± 25	319 ± 18
QTp_Fridericia_ (ms)	306 ± 20	260 ± 24	262 ± 21	283 ± 17	294 ± 20	292 ± 24	296 ± 15
QTp_Lilly_ (ms)	308 ± 20	276 ± 25	274 ± 23	294 ± 18	304 ± 21	301 ± 24	305 ± 16
QTp_Framingham_ (ms)	311 ± 19	284 ± 18	284 ± 17	300 ± 15	308 ± 17	307 ± 20	310 ± 13
Baseline							
Te (ms)	93 ± 14**	72 ± 15	86 ± 35	77 ± 7	68 ± 14	72 ± 8	76 ± 22
Te_Bazett_ (ms)	99 ± 15	114 ± 27*	125 ± 56	103 ± 13	87 ± 17	92 ± 12	98 ± 29
Te_Fridericia_ (ms)	97 ± 14	98 ± 22*	110 ± 48	93 ± 9	80 ± 16	85 ± 9	90 ± 26
Te_Lilly_ (ms)	98 ± 14	105 ± 24*	116 ± 51	97 ± 11	83 ± 16	87 ± 11	93 ± 27
Te_Framingham_ (ms)	110 ± 29	164 ± 19*	164 ± 42	141 ± 25	128 ± 19	130 ± 23	134 ± 32
Testosterone							
Te (ms)	52 ± 12**	52 ± 12	71 ± 9	71 ± 13	72 ± 11	72 ± 12	64 ± 12
Te_Bazett_ (ms)	110 ± 78	81 ± 17*	98 ± 15	93 ± 15	92 ± 15	90 ± 20	80 ± 14
Te_Fridericia_ (ms)	108 ± 76	70 ± 15*	88 ± 12	85 ± 14	85 ± 14	85 ± 10	74 ± 13
Te_Lilly_ (ms)	109 ± 77	74 ± 16*	92 ± 13	88 ± 14	88 ± 14	86 ± 18	76 ± 13
Te_Framingham_ (ms)	121 ± 75	142 ± 11*	144 ± 15	135 ± 12	135 ± 16	127 ± 21	117 ± 13

These data are detected manually with tangential method. **p < 0.001 or *p < 0.05 baseline versus testosterone

*RR* RR interval, *QTe* QT end, *QTp* QT peak, *Te* T end

**Table 6:** QTe, QTp and Te Variability and Coherence Data after testosterone therapy

Variables	Baseline subjects *N* = 13	Testosterone subjects *N* = 13	*P* values
Rest			
QTe mean (ms)	358 ± 33	349 ± 30	Ns
QTe variance (ms^2^)	44 [30]	48 [47]	Ns
QTe standard deviation	7 ± 2	7 ± 1	Ns
RR (ms)	878 ± 207	925 ± 177	Ns
RR variance (ms^2^)	1090 [1950]	788 [1663]	Ns
RR standard deviation	37 ± 21	39 ± 22	Ns
QTeVI	− 0.75 [0.77]	− 0.52 [0.62]	Ns
RR → QTe, coherence	0.206 ± 0.034	0.215 ± 0.029	Ns
QTp mean (ms)	265 ± 23	253 ± 25	Ns
QTp variance (ms^2^)	27 [26]	27 [16]	Ns
QTp standard deviation (ms^2^)	5 ± 1	5 ± 1	Ns
QTpVI	− 0.77 [0.55]	− 0.50 [0.79]	Ns
RR → QTp, coherence	0.236 ± 0.036	0.212 ± 0.034	Ns
Te (ms)	94 ± 14	96 ± 9	Ns
Te variance (ms)	42 [46]	74 [40]	Ns
Te standard deviation	8 ± 2	8 ± 1	Ns
TeVI	0.60 [0.43]	0.71 [0.47]	Ns
RR → Te, coherence	0.220 ± 0.038	0.208 ± 0.042	Ns
QTp → Te, coherence	0.471 ± 0.061	0.453 ± 0.068	Ns
10 min exercise recovery			
QTe mean (ms)	283 ± 27	285 ± 18	Ns
QTe variance (ms^2^)	324 [448]	369 [319]	Ns
QTe standard deviation	23 ± 7	19 ± 6	Ns
RR (ms)	562 ± 130	583 ± 71	Ns
RR variance (ms^2^)	2612 [2859]	3431 [4834]	Ns
RR standard deviation	65 ± 41	64 ± 24	Ns
QTeVI	− 0.28 [0.54]	− 0.36 [0.26]	Ns
RR → QTe, coherence	0.235 ± 0.055	0.219 ± 0.025	Ns
QTe-RR slope	0.35 [0.23]	0.34 [0.12]	Ns
QTp mean (ms)	206 ± 20	213 ± 20	Ns
QTp variance (ms^2^)	291 [311]	293 [549]	Ns
QTp standard deviation (ms^2^)	19 ± 6	20 ± 7	Ns
QTpVI	− 0.12 [0.44]	− 0.20 [0.24]	Ns
RR → QTp, coherence	0.218 ± 0.031	0.216 ± 0.024	Ns
QTp-RR slope	0.30 [0.13]	0.31 [0.11]	Ns
Te (ms)	76 ± 10	75 ± 9	Ns
Te variance (ms)	106 [95]	82 [87]	Ns
Te standard deviation	11 ± 2	10 ± 3	Ns
TeVI	0.22 [0.79]	0.16 [0.87]	Ns
RR → Te, coherence	0.208 ± 0.048	0.200 ± 0.016	Ns
QTp → Te, coherence	0.417 ± 0.037	0.447 ± 0.095	Ns
Te-RR slope	0.077 [0.09]	0.002 [0.102]	0.028

These data are detected automatically with a custom software. Values are expressed as mean ± SD or median [interquartile range 75th percentile–25th percentile]

## Discussion

The present study primarily confirms that corrected QTe and QTp at rest are longer in the hypogonadal subjects than age-matched normal controls. This finding confirms many other previous studies where it has been stated that the reduction or absence of testosterone levels prolongs the repolarization phases with a possible proarrhythmic effect [35–38]. Interestingly, in hypogonadals patients, we found that during the post-exercise recovery phase the QTe interval, corrected for the heart rate using all the available formulas except of the Bazett one [39–41], was longer and, contextually, there was significantly lower QTp → Te coherence. In fact, it has been previously demonstrated that the cubic root equation (Fridericia’s) might be more accurate than the square root (Bazett’s) or several complex formulas for correcting measured QT intervals for cardiac cycle length in middle-aged men [41]. Furthermore, the instantaneous QTp, obtained by the standard method (i.e. measuring three consecutive RR and the following QRS-T intervals), was longer at rest and in three over the six measurements of the recovery phase (3rd, 5th, 9th minute) regardless of the heart rate. Finally, since any differences of corrected Te both at rest or during the recovery phase was found, it is reasonable that the longer QTe was caused by an abnormal first part of the repolarization (i.e. QTp). Thus, our data suggest that the whole repolarization phase was altered by an abnormal duration of first part of QT at low level of heart rate and sympathetic activity. In this context, it might be possible to find a similarity between the effect of low level of testosterone and the congenital long QT type 3 syndromes (due to Na channel SN5) [42] where the arrhythmic events usually occur during the night at lower sympathetic activity and heart rate. Leptin increases sympathetic nerve activity in humans [43]; even if we did not evaluate leptin variations upon TRT, the maintenance of stable BP in our patients might have been explained by leptin reduction and amelioration of insulin sensitivity that usually occurs after short-term TRT in severe hypogonadal patients with metabolic syndrome [34, 44].

Indeed, the action potential duration of cardiac cells, corresponding to the surface QTe interval, is maintained by the perfect temporal activation and inactivation of the sodium, calcium and potassium current by means of specific ionic channels. Testosterone is able to shorten the action potential duration and, namely the QTe, especially influencing the L-type calcium channel (*I*_CaL_) and slow delayed rectifier K^+^ channel (I_Ks_). Accordingly, testosterone could be able to reduce the QTe length throughout an inhibition of the *I*_CaL_ and an increase of the *I*_Ks_ activities [42, 45–48], thus decreasing the calcium entry and increasing the potassium efflux. Consequently, we hypothesized that the absence of androgens in the hypogonadal subjects induces an increase of QTe by means of an increase of calcium influx and a lower action on specific ion channels [49].

Most of the myocardial temporal dispersion variables were similar between controls and hypogonadal patients both at rest and during the recovery phase. A possible explanation could be that these QT dynamic data are important to explain severe cardiac events [12] but they were not enough sensitive in subjects with almost normal cardiac function and, hence, a low relative risk of malignant ventricular arrhythmias. Thus, we hypothesize that, in case of further repolarization reserve reduction (myocardial ischemia, hypertrophy, heart failure, hypokalemia, genetic polymorphism of ionic channel etc.…) [49–51] also in a hypogonadal patient, these ECG parameters could be useful in disclosing an increased SCD risk.

The QTp → Te coherence during the recovery phase was the only myocardial temporal dispersion variable found to be different in hypogonadal patients with respect to the controls. The coherence between two oscillatory components expresses a strong linear coupling between QTp-Te interval fluctuation and the ability of two signals to have similar behavior in the time. We previously observed that, at rest, a reduction of QTp → Te coherence was associated with sustained ventricular tachycardia in patients with low ejection fraction and chronic heart failure [25]. In the present study, this parameter was altered (i.e. reduced) solely during the recovery phase, condition characterized by an autonomic nervous system imbalance with still high sympathetic activity. The underlying mechanisms for altering QTp → Te coherence are unknown and controversial [52–54]. Undoubtedly, QTp and Te have two different electrophysiological meanings. In some studies, QTp could be referred to action potential duration of the epicardial layer [55, 56]; on the contrary, Te predominantly is influenced by the M-cell layer repolarization and this last layer also showed the longer depolarization duration. Thus, these authors consider the Te reflecting the maximum difference in repolarization between the myocardial layers. For this reason, they have suggested it as a non-invasive marker of transmural dispersion of repolarization [54, 55]. Therefore, Te depends on *I*_Kr_, *I*_Ks_ and *I*_K1_ function, whereas the QTp reasonably is influenced by the depolarization phase, so by Na currents, and by the early repolarization phase, both, mainly under the *I*_to_ control and by the sarcoplasmic reticulum Ca uptake (up) currents [17, 56, 57]. Definitely, the dysfunction of these ion channels’ network could alter one of these two QT segments, reducing their coherence and probably, increasing the ventricular arrhythmias risk’s [17, 57], especially during sympathetic stress. Given the abovementioned mechanisms, albeit merely speculative, the sympathetic stress might have increased the sensitivity of QTp → Te coherence so that low level of this parameter could be indicative of an intermediate risk of malignant ventricular arrhythmias.

The TRT in hypogonadal subjects seemed to have a weak effect on the duration and on the dynamic of repolarization phases. Indeed, after 6 months of therapy we did not have any difference of corrected QT in baseline and during exercise recovery. Although, in vitro, some of our previous studies reported a reduction of action potential duration in cardiac cells, few data existed on QT in the hypogonadal patients. In regard to the corrected QT, Charbit et al. found a reduction of 13.6 ± 2.8 ms between low and high levels of serum testosterone (low versus high testosterone level medians: 6 versus 52.6 nmol/L) [58]. On the contrary, Pecori Giraldi et al. did not find a reduction of corrected QT except for a small percentage with an abnormal corrected QT (> 440 ms) [36–38]. In our study, the level of testosterone reached was less than half of the Charbit study and none of our hypogonadal subjects had a corrected QT at rest higher of 440 ms; thus, we cannot make a definitive comparison with the previous cited studies. However, although our patients were older than the Pecori Giraldi study (for these reasons, we reported lower testosterone serum levels), we obtained the similar results with a corrected QT at rest unmodified by the TRT. On the contrary, other authors reported an improvement of QTpVI in hypogonadal subjects with spinal cord injury during TRT [59, 60]. Probably, the study is incomparable because our hypogonadal subjects were 20 years older than the previous patients of abovementioned study. Only corrected Te and Te-RR slopes (resulted from Te/RR relation) were significantly reduced after the TRT, the first one at the peak and the second during whole recovery phase. The reduction of corrected Te was consistent with the possible reduction malignant arrhythmias risk. The decrease of the Te-RR slope indicates a reduction of steepness of the regression line obtained for these two variables. Given that, a high steepness represents a major risk of sudden death in heart failure [14, 30–33]; therefore, the observed decrease of Te-RR slope might hypothetically support a possible reduction of the SCD risk.

One important study limitation consists in the operator-dependent evaluation of single patient EKGs may be considered weak; indeed, in our prior experience, this single-operator in-deep analysis led us to identify any important variation related to myocardial dispersion and repolarization to better predict QT variations [41]. Another limitation is represented by the limited number of subjects studied; we acknowledge the great ethical difficulties to maintain any hypogonadal subject without TRT and also for this reason we did not enrol a placebo-controlled treated group that was not permitted by our Ethical Committee. We tried to overcome this bias by using a control group in whom no treatment for hypogonadism was indicated. Finally, we recognize that expected changes in symptoms and signs of hypogonadism as well as in hormonal and body composition patterns are not presented since they were not in the aim and scope of the present study; they had been already reported in previous studies [34, 61].

In conclusion, an increase of QT duration not heart-rate related in hypogonadal patients has been observed, but most of the dynamic markers of myocardial temporal dispersion of repolarization were not altered neither at rest or during the post-exercise recovery phase. Therefore, it is likely that hypogonadism per se does not increase the risk of malignant ventricular arrhythmias. Nevertheless, some subtle modifications in the repolarization phase either at rest (stable QT prolongation) or during autonomic nervous system unbalance (reduced QTp → Te coherence during the recovery phase), as well as the improvement of some ECG variables after TRT (Te, Te/RR slope) might support the idea of a leading tendency to ventricular arrhythmias in hypogonadal patients and, hence, it claims for a close control of possible other conditions able to further reduce the repolarization reserve. Hypogonadal patients are generally considered at increased risk of major adverse cardiovascular events and sudden cardiac death. When considering the results of our EKG study, it seems appropriate to treat severe hypogonadism with TRT independently of age and comorbidities, preceding it by a thorough cardiologic counselling to avoid possible ventricular adverse event (QT) related to testosterone action on the repolarization phase.
